# Survival After Cardiac Arrest Due to an Anomalous Right Coronary Artery in a Young Athlete

**DOI:** 10.1016/j.jaccas.2025.104122

**Published:** 2025-08-13

**Authors:** Jorge A. Silva-Estrada, Melissa Gastelum-Bernal, Héctor Díliz-Nava, Karina Coronado-Centeno, Carlos González-Rebeles, Pérez-Juárez Fabiola, Itzel Ríos-Olivares, Jair Rafael Osorio-Ugarte, Carlos Corona-Villalobos, Alexis Palacios-Macedo

**Affiliations:** a Department of Cardiology, Instituto Nacional de Pediatría, Mexico City, Mexico; b Department of Cardiovascular Surgery, Instituto Nacional de Pediatría, Mexico City, Mexico

**Keywords:** cardiac magnetic resonance, coronary vessel anomaly, ventricular fibrillation

## Abstract

**Background:**

A 15-year-old Mexican boy experienced sudden cardiac arrest (SCA) during physical exertion.

**Case Summary:**

The patient received bystander cardiopulmonary resuscitation. An anomalous aortic origin of the right coronary artery was diagnosed by echocardiography. Extracorporeal membrane oxygenation was initiated for cardiogenic shock. Coronary unroofing was performed. Results of stress perfusion cardiac magnetic resonance imaging were negative for ischemia; myocardial infarction was ruled out. The patient recovered without neurological sequelae.

**Discussion:**

Anomalous coronary arteries are a leading cause of SCA in young athletes. There is a gap in evidence regarding indications for implantable cardioverter-defibrillator implantation in surgically corrected coronary arteries.

**Take-Home Messages:**

Anomalous origin of the coronary arteries causing SCA does not always cause myocardial infarction, leaving chronic ischemia as a possible mechanism for cardiovascular events. Stress perfusion imaging and electrophysiologic consultation should be carried out in following up these patients. An appropriate multidisciplinary approach that can result in excellent outcomes is achievable even in developing countries.

## Graphical Abstract

**Figure:**
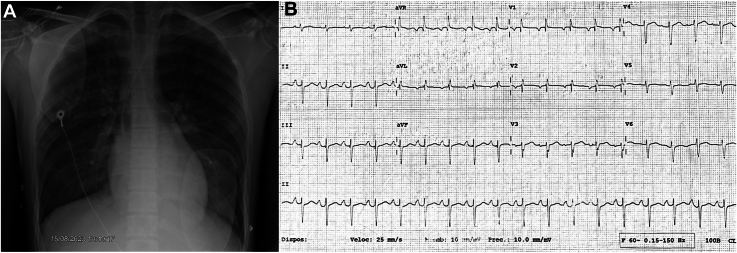

**Take-Home Messages**

• Anomalous origin of the coronary arteries causing SCA does not always cause myocardial infarction, leaving chronic ischemia as a possible mechanism for cardiovascular events.
• Stress perfusion imaging and electrophysiologic consultation should be carried out in following up these patients.
• An appropriate multidisciplinary approach that can result in excellent outcomes is achievable even in developing countries.

## History of Presentation

A 15-year-old Mexican boy experienced cardiac arrest while sprint racing. His coach witnessed his sudden collapse and called a staff nurse who was supervising a summer camp at the same running track. Both immediately started cardiopulmonary resuscitation (CPR). Within <8 minutes, an ambulance took him to the children's hospital where the nurse worked, which was fortuitously the closest tertiary care hospital. During this time, he received chest compressions and rescue breaths (30:2) and arrived at the emergency department, where he received 200-J defibrillation after ventricular fibrillation was picked up.

## Past Medical History

The patient was a previously healthy competitive swimmer. He had been doing strenuous physical activity for the past 5 years without any exertional symptoms. There was no family history of cardiovascular or genetic disorders.

## Differential Diagnosis

The patient received CPR at the emergency department for approximately 30 minutes, with advanced airway management. Several doses of adrenaline were administered alongside 3 200-J shocks for defibrillation and 2 70-J cardioversions because of alternating ventricular tachycardia and ventricular fibrillation episodes.

Urgent cardiology consultation was requested to rule out causes of sudden cardiac arrest (SCA) such as cardiomyopathies, coronary anomalies, channelopathies, and congenital heart diseases. Due to cardiogenic shock and refractory arrhythmias, extracorporeal membrane oxygenation (ECMO) was initiated.

## Investigations

**Figure 1:**
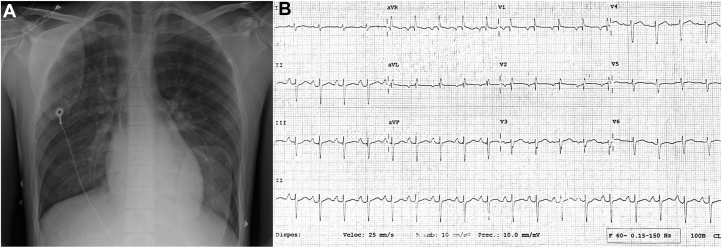
Chest Radiograph and 12-Lead Electrocardiogram (A) Anteroposterior projection shows mild venocapillary congestion. (B) Electrocardiogram in sinus rhythm with a normal QT interval. A Brugada pattern was ruled out; unspecific flattened T waves in leads V_1_ and V_2_. Left anterior fascicular block. Criteria for left atrial enlargement were met: a notched P-wave in lead DII and a widened negative P-wave in lead V_1_.

**Figure 2:**
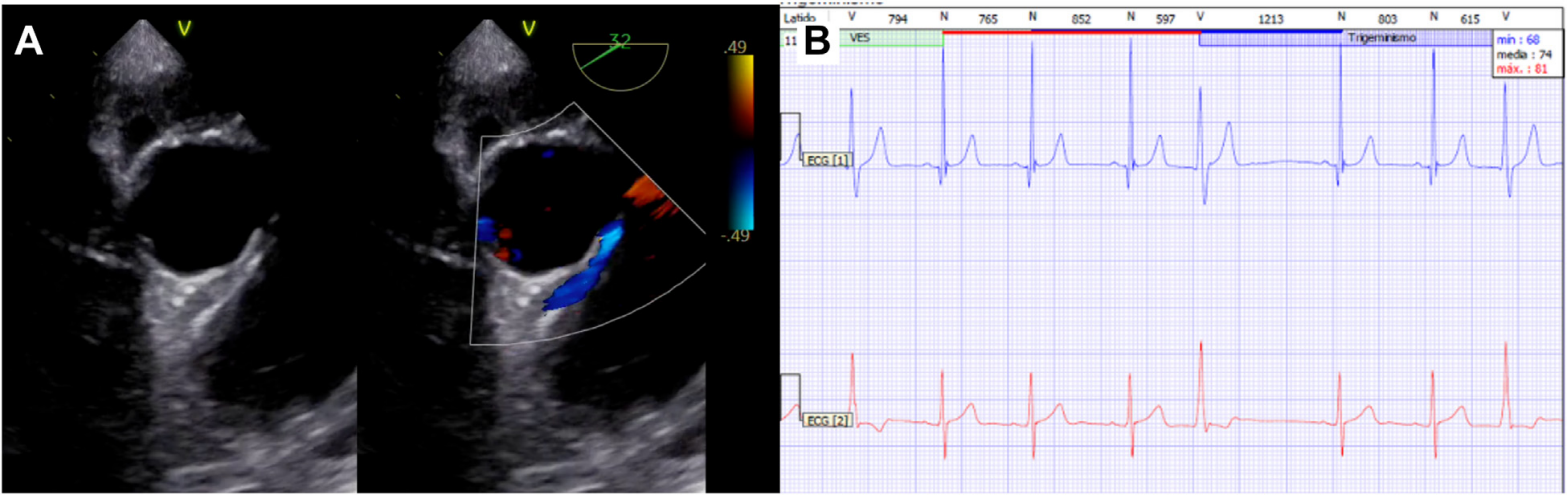
Transesophageal Echocardiogram and Holter Monitoring (A) Midesophageal short-axis view shows anomalous origin of the right coronary artery from the left aortic sinus with an interarterial course; the fixed narrow coronary lumen strongly suggests an intramural segment. (B) 24-hour Holter monitoring registered 8% monomorphic premature ventricular contractions, in isolation and trigeminy.

There was evidence of troponin leakage and increased serum brain natriuretic peptide (troponin I 24 ng/mL and pro–brain natriuretic peptide 2,540 pg/mL). Initial anteroposterior chest radiography showed mild venocapillary congestion, and rest electrocardiography did rule out long-QT interval and Brugada pattern (Figure 1). Transthoracic echocardiography revealed dilated and severely hypokinetic ventricles, with a left ventricular ejection fraction (LVEF) of 30%. Twenty-four hours after ECMO cannulation, transesophageal echocardiography demonstrated anomalous aortic origin of the right coronary artery with an interarterial course, with a high probability of an intramural segment (Figure 2A, Video 1). Twenty-four-hour Holter monitoring reported a few episodes of sinus tachycardia and monomorphic premature ventricular contractions (Figure 2B).

## Management

**Figure 3:**
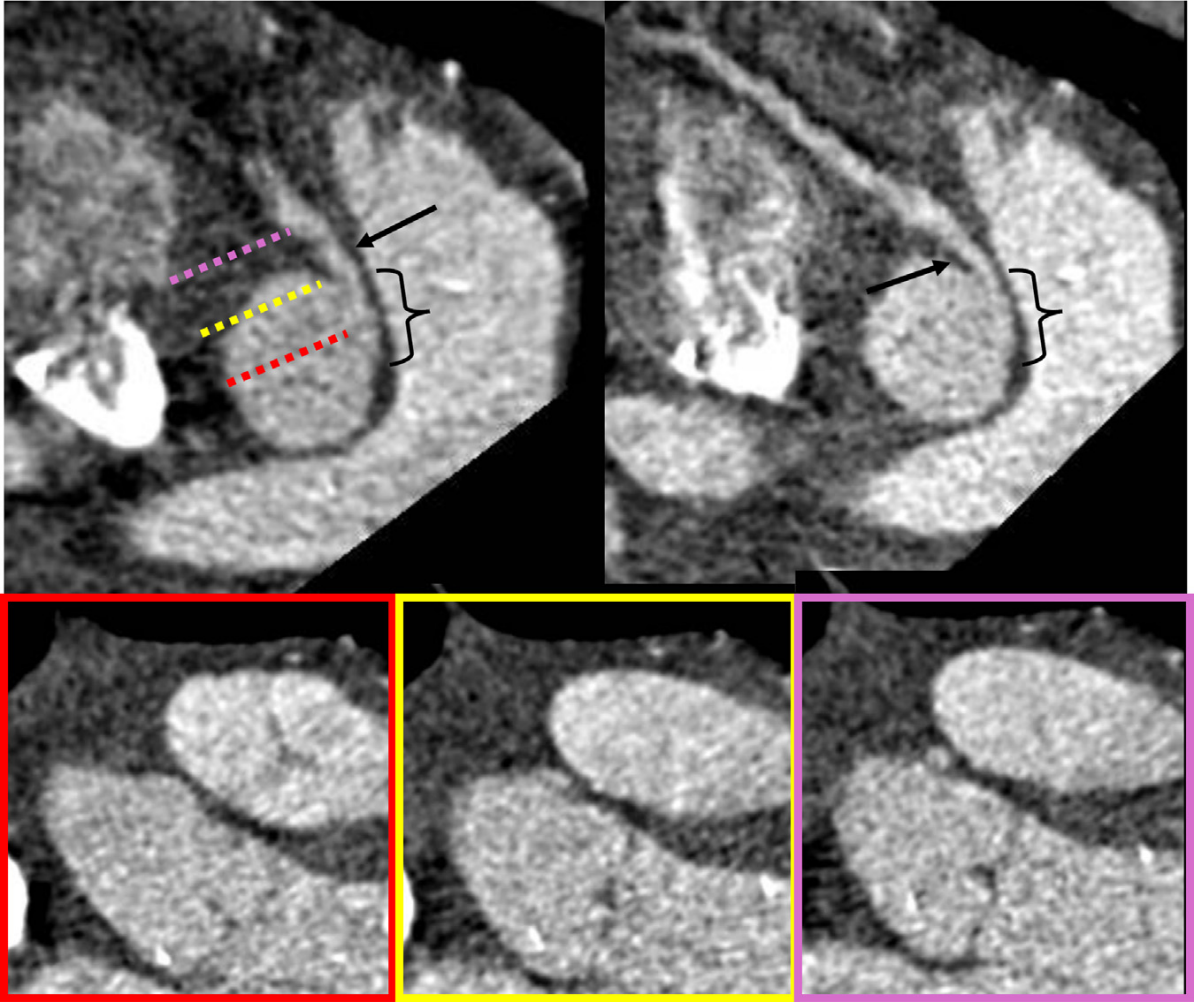
Postoperative Coronary Computed Tomographic Angiography Double-oblique sagittal view and vessel short-axis views show a right coronary artery with a 9-mm unroofed segment (brackets). There is persistent angulation of <40° at the exit site and an interarterial course distal to the neo-ostium of 4 mm (arrow). Originally, coronary ostium was close to the intercoronary commissure and above the sinotubular junction, and nomenclature according to the Leiden convention and Texas Children's group was 2R∗, LCx and 2a-IV, respectively.

**Figure 4:**
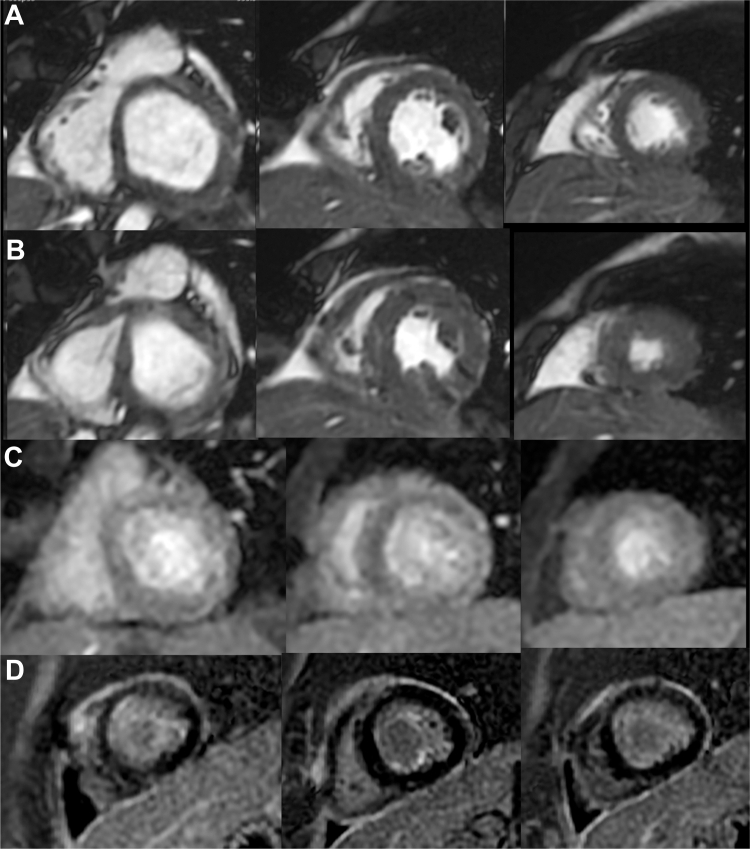
Rest Perfusion Cardiac Magnetic Resonance Short-axis images. Cine images in diastole (A) and systole (B) where generalized left ventricular hypokinesis was observed, predominantly at the interventricular septum and mild pericardial effusion. Rest left ventricular ejection fraction of 40%. (C) During first-pass perfusion at rest with normal myocardial enhancement. (D) Late gadolinium enhancement images show myocardium homogeneously hypointense, ruling out myocardial infarction.

For venoarterial ECMO, surgeons decided to carry out central cannulation to achieve direct left ventricular venting. Surgical unroofing of the intramural segment was performed 7 days after diagnosis. Coronary computed tomographic angiography (CTA) was requested after surgery. Preoperative CTA could not be ordered, because of inability to mobilize the patient to the computed tomography suite: the hospital elevators could not fit an intensive care bed plus the ECMO machine. Postoperative coronary CTA showed the anomalous right coronary artery emerging just above the left coronary sinus commissure with an inter-arterial course and an unroofed intramural segment of 9 mm (Figure 3). The left main coronary artery and anterior descending and left circumflex branches were normal. The patient spent 4 weeks in the cardiovascular intensive care unit, where he received a tracheostomy after 2 failed attempts at extubation and a gastrostomy. He had multiple episodes of ventricular tachycardia and fibrillation that required lidocaine infusion. Serial echocardiography showed a slight improvement of LVEF up to 35%. In the intensive care unit, he was weaned from mechanical ventilation. Neurologically, he had only mild weakness at the 4 extremities. Cardiac magnetic resonance (CMR) with a rest perfusion protocol was done 8 weeks after hospital admission and showed no evidence of myocardial infarction and an LVEF of 40% (Figure 4, Video 2). After consultation with the electrophysiology team, an implantable cardioverter-defibrillator (ICD) was placed just before discharge. He was discharged on carvedilol, amiodarone, enalapril, spironolactone, furosemide, and acetylsalicylic acid. Four months later, the patient returned for an exercise stress test and myocardial perfusion imaging following our institution's CMR dobutamine stress protocol (Video 3). There was no evidence of ischemia. A heart rate of 180 beats/min and a rate-pressure product of 24,000 were achieved with a dobutamine dose of 40 μg/kg/min. At rest (heart rate 70 beats/min), septal hypokinesis was observed, whereas at maximum stress, there were no perfusion defects, and we documented normal segmental wall motion with preserved systolic thickening. The patient received clearance for moderate leisure-time exercise after finishing an 8-week cardiac rehabilitation program.

## Discussion

The incidence of sudden cardiac death (SCD) in athletes is 0.5 to 1 per 100,000 patient-years, and SCD contributes to 16% of deaths among athletes. Coronary anomalies are found in 19% of these cases and stand out as the second leading cause of SCD. Survival after out-of-hospital SCA is poor, with survival rates as low as 9% to 16%.^1^^,^^2^

Anomalous right coronary artery from the left aortic sinus reaches a prevalence of 0.28% to 0.92%, up to 10 times the prevalence of anomalous left coronary arteries. According to the largest cohorts, anomalous right coronary arteries carry less risk for SCD or SCA; however, cardiovascular events are still reported in those patients with high-risk features.^3^ These high-risk features need to be thoroughly assessed using multimodality imaging and include an interatrial course, a long intramural course, a slitlike ostium, an acute takeoff angle (<45°), and evidence of inducible ischemia on functional stress testing.^4^

The phenotypic features of our patient rendered him a good candidate for unroofing of the intramural segment, as recommended in a recent study from the Congenital Heart Surgeons Society.^5^ Through unroofing, this technique directly addresses the intramural compression and even allows the creation of an enlarged neo-ostium in the correct sinus of Valsalva. One of the relative contraindications of this technique is the position of intramural course below the level of commissure insertion, the presence of a prominent intercoronary pillar, and a short intramural segment (<5 mm).^6^ It is important to bear in mind that an acute takeoff angle at the neo-ostial level might persist and can contribute to low coronary flow and ischemia, even after surgery.

There are 2 main reasons to perform functional studies to assess inducible ischemia in patients with a high-risk anomalous right coronary artery from the left aortic sinus: in asymptomatic patients, to decide whether to offer surgical intervention, and in patients who underwent surgery, to help stratify the risk for future cardiovascular events and guide exercise restrictions.^7^^,^^8^

A gap in evidence exists regarding recommendations for ICD implantation in patients with at-risk coronary artery anomalies who undergo surgical unroofing or other surgical correction.^9^ A few case reports and expert commentaries support a case-by-case approach, with special focus on high-risk features such as recurrent ventricular arrhythmias, evidence of myocardial fibrosis or infarction, and inducible ischemia.^10^

## Follow-Up

Serial echocardiography and noncontrast CMR showed improved LVEF. Eighteen months after the event, an LVEF of 48% was reported. Twenty-four-hour Holter monitoring 6 months after discharge reported normal sinus rhythm. The patient remains asymptomatic and tolerates exercise, such as jogging and swimming. His tracheostomy cannula was removed shortly after discharge, and the gastrostomy was removed 2 months afterward. Eighteen months after initial cardiac arrest, he had no neurologic sequelae and attended school and recreational activities normally.

## Conclusions

Coronary artery anomalies are a leading cause of SCA in previously asymptomatic young athletes, even in those who have long histories of competitive training. Despite immediate initiation of CPR, survival rates remain low, especially for out-of-hospital cardiac arrests in developing countries. In our patient, immediate bystander CPR and expedited ambulance transport to a tertiary care children's facility with the ability to offer ECMO and coronary unroofing were crucial for survival. Even though acute ischemia due to vessel compression is the most accepted mechanism of cardiac arrest, it is important to note that some patients do not develop myocardial infarction, leaving room for other theories, such as adverse myocardial remodeling after chronic ischemia as a substrate for arrhythmogenic events. Despite surgical intervention and negative results on stress perfusion tests, the suitability of an ICD implantation must be assessed by electrophysiology experts in all patients because of the potential risk for ongoing arrhythmias and even SCD.

## Funding Support and Author Disclosures

The authors have reported that they have no relationships relevant to the contents of this paper to disclose.

## Appendix

Video 1 Interarterial course of anomalous right coronary artery.

Video 2 Rest perfusion CMR did not reveal evidence of infarction, with a reduced LVEF of 40%.

Video 3 Stress perfusion CMR with dobutamine was negative for ischemia. There were no perfusion defects, with improved wall motion in all segments at maximum stress.
